# Sheep breed-specific response to environment challenge against *Haemonchus contortus* and effect on immuno-hematological parameters

**DOI:** 10.1007/s11259-026-11304-2

**Published:** 2026-06-06

**Authors:** Cintia Hiromi Okino, Hornblenda Joaquina Silva Bello, Simone Cristina Méo Niciura, Amanda Freitas da Cunha, Estevão Camilo da Costa, Emanuelle Martins de Campos, Rafaela Tami Ikeda Kapritchkoff, Lorrane Gabriele Pinheiro Corrêa, Alessandro Pelegrine Minho, Sérgio Novita Esteves, Ana Carolina de Souza Chagas

**Affiliations:** 1 Embrapa Pecuária Sudeste, Rodovia Washington Luiz, Km 234 s/n, Fazenda Canchim, PO Box 339, São Carlos, SP 13560-970 Brazil; 2 Centro Universitário Central Paulista (UNICEP), Rua Miguel Petroni, São Carlos, SP 5111, 13563-470 Brazil; 3 Centro de Ciências Biológicas e da Saúde, Universidade Federal de São Carlos (UFSCAR), Rodovia Washington Luiz km 235, PO Box 676, São Carlos, SP 13565-905 Brazil; 4 Faculdade de Ciências Agrárias e Veterinárias, Universidade Estadual Paulista (UNESP), Rodovia Prof. Paulo Donato Castellane, Jaboticabal, SP 14884-900 Brazil; 5 Faculdade de Ciências Agrárias e Tecnológicas, Universidade Estadual Paulista (UNESP), Rodovia Comandante João Ribeiro de Barros km 651, Dracena, SP 17900-000 Brazil

**Keywords:** hemogram, ovine, abomasum, *Haemonchus contortus*, gastrointestinal nematodes, Santa Inês, White Dorper, Texel

## Abstract

Variable resistance to *Haemonchus contortus* infection and the associated immune mechanisms have been investigated and characterized in different sheep breeds adapted to tropical climates. In a recent study, we compared the local immune responses of the resistant Santa Inês with those of the susceptible White Dorper and Texel sheep breeds under chronic haemonchosis, and evaluated the correlations of immune-related transcripts and different parasitic stages and sexes. The aim of present study was to further elucidate the immune mechanisms among these sheep breeds by comparing local responses, microscopic lesions in the abomasum and hematological parameters in lambs raised under varying levels of parasitic challenge. The naturally resistant Santa Inês breed maintained similar phenotypic characteristics across different levels of parasitic infection loads, whereas White Dorper and Texel breeds exhibited increased susceptibility under moderate to high challenge. Texel lambs showed altered mean corpuscular volume, indicative of macrocytic anemia, and also displayed the lowest resistance under low challenge conditions. Significantly higher levels of eosinophils, lymphocytes and monocytes in peripheral blood, along with increased expression of *Galectin-11* gene in the abomasum, were observed in Santa Inês sheep compared to the other breeds. These responses may represent key host mechanisms contributing to improved control of infection. Overall, our findings demonstrate that resistance to *H. contortus* infection is breed-dependent and modulated by environmental parasite challenge, providing insights that may forward to the targeted strategies for sheep flock management.

**Supplementary Information:**

The online version contains supplementary material available at https://doi.org/10.1007/s11259-026-11304-2.

## Introduction

*Haemonchus contortus*, a gastrointestinal nematode (GIN), is considered one of the most pathogenic parasites affecting sheep and is frequently associated with the development of severe anemia, with major occurrence in tropical or subtropical climates (Arsenopoulos et al. 2021). Widespread anthelminthic resistance has been reported for this parasite, and currently no commercially available drug is able to completely clear the infection (Bassetto et al. 2024). In this sense, alternative control strategies, such as immune-prophylaxis and genetic selection of resistant animals, may help reduce the spoliation induced by this GIN (Amarante et al. 2004; Louvandini et al. 2006).

Parasitic resistance generally encompasses both resistance to infection and resistance to the consequences of infection, implicating into tolerance or resilience to parasitism. As reviewed by Bishop (2012), resistance is defined as the host´s ability to control the parasite life cycle, including the establishment of ingested larvae, parasite development within the host, and parasite mortality and fecundity, and consequently fecal egg counts. On the other hand, parasitic tolerance refers to the host´s ability to withstand the pathogenic effects of infection, whereas resilience is closely related to tolerance and describes the capacity to maintain performance despite disease.

Differential resistance and/or resilience to parasitic infections is well documented among different sheep breeds. Hair-type animals, such as Santa Inês, Morada Nova and Red Maasai breeds, exhibit naturally higher resistance or resilience to *H. contortus* compared to wool or semi-wool types, including Ile de France, Suffolk and White Dorper (Mugambi et al. 1997; Wanyangu et al. 1997; Amarante et al. 2004; Toscano et al. 2019; Kapritchkoff et al. 2024; Chagas et al. 2024). In this sense, resistance to *H. contortus* has been shown to increase from White Dorper to Texel and then to Santa Inês (Kapritchkoff et al. 2024; Okino et al. 2025). Distinct local responses are elicited during infection. A strong innate response mediated by *TLR2* has been observed in both Santa Inês and White Dorper breeds, whereas an enhanced Th2-type response was identified in Texel (Okino et al. 2025). Additionally, counts of *H. contortus* developmental stages were correlated with local gene expression levels, with *TLR2*-associated activity linked to early parasite stages and complement system activity associated with later stages (Okino et al. 2025).

Beyond differences in resistance or resilience, environmental levels of parasitic contamination may also be handled differently among sheep breeds. In this context, the parasitic burden in pasture is directly influenced by the density of infected animals and by the proportion of susceptible or resistant individuals within the flock. Therefore, to further characterize resistance and resilience among sheep breeds and the associated immune mechanisms, this study evaluated lambs from three breeds (Santa Inês, White Dorper and Texel) under variable loads of natural infection with *H. contortus*. We compared immuno-hematological kinetics among these breeds raised in paddocks with varying animal densities and breed distribution.

## Materials and methods

### Experimental design

One-hundred fifty-one lambs (White Dorper: 23 females and 19 males, Texel: 15 females and 29 males, Santa Inês: 33 females and 32 males) from the Embrapa Pecuária Sudeste experimental farm born between July to September 2024 were used for study.

The lambs were raised together in an area endemic for *H. contortus*, distributed across three adjacent 4-hectare paddocks with differing breed distribution and stocking density (Table 1). In terms of stocking density, paddock A had 7 (14.58%) more lambs than paddocks B and C paddocks (13.75 vs. 12 AU/ha). Regarding breed-related resistance, paddock C had the highest proportion of the most resistant breed (Santa Inês), whereas paddock A had the lowest proportion and paddock B showed intermediate values. Consequently, the paddocks were classified as high (A), intermediate (B) and low (C) challenge for parasitic infection.

**Table 1:** Distribution and frequency of experimental lambs per breed, paddocks, sex and number of male lambs euthanized submitted to worm counts, histopathology and gene expression quantification

Paddock	Breed	Female	Male	Female + male	Euthanasia*
A	White Dorper	12/55 (21.82%)	7/55 (12.73%)	19/55 or 1.2 (34.55%)	3/30 (10%)
(high challenge)	Texel	4/55 (7.27%)	12/55 (21.82%)	16/55 or 1.0 (29.09%)	4/30 (13.3%)
	Santa Inês	10/55 (18.18%)	10/55 (18.18%)	20/55 or 1.25 (36.36%)	2/30 (6.67%)
	Overall	26/55 (47.27%)	29/55 (52.27%)		10/30 (33.3%)
B	White Dorper	6/48 (12.50%)	7/48 (14.58%)	13/48 or 1 (27.08%)	5/30 (16.67%)
(intermediate challenge)	Texel	6/48 (12.50%)	7/48 (14.58%)	13/48 or 1 (27.08%)	2/30 (6.67%)
	Santa Inês	11/48 (22.92%)	11/48 (22.92%)	22/48 or 1.7 (45.83%)	4/30 (13.3%)
	Overall	23/48 (47.91%)	25/48 (52.08%)		11/30 (36.7%)
C	White Dorper	5/48 (10.42%)	5/48 (10.42%)	10/48 or 1 (20.83%)	2/30 (6.67%)
(low challenge)	Texel	5/48 (10.42%)	10/48 (20.83%)	15/48 or 1.5 (31.25%)	4/30 (13.3%)
	Santa Inês	12/48 (25.00%)	11/48 (22.92%)	23/48 or 2.3 (47.92%)	3/30 (10%)
	Overall	22/48 (45.83%)	26/48 (54.16%)		9/30 (30%)

* All euthanized lambs were males and genotyped as BB homozygous for the β−globin haplotype

All lambs were weaned at 84 days of age and monitored for fecal egg counts (FEC) (Ueno and Gonçalves 1998), packed cell volume (PCV) and complete hemogram at 105 and 189 days old (D105 and D189). At approximately 210 days old, 10 males from each sheep breed were euthanized and subjected to necropsy. Based on our previous studies (Okino et al. 2021a, b, 2023, 2024; Kapritchkoff et al. 2024), in which the β-globin haplotype was significantly associated with variation in resistance to *H. contortus* infection, all experimental lambs were genotyped. However, only two lambs carried the AA haplotype (one Texel and one White Dorper), while 45 lambs carried the AB haplotype (7 White Dorper, 1 Texel and 32 Santa Inês). Due to this unbalanced distribution across haplotypes and breeds, the β-globin haplotype could not be included as between-subject factor. Consequently, only lambs harboring the BB haplotype were selected for euthanasia (Okino et al. 2021b). All procedures were approved by the Embrapa Pecuária Sudeste Ethical Committee for Animal Experimentation (process n. 02/2022), in accordance with ethical principles and guidelines of animal experimentation adopted by the Brazilian College of Experimentation.

### Hematological analysis and fecal egg counts

Blood samples collected in EDTA vacutainer tubes were analyzed using the microhematocrit method for PCV determination and subjected to complete hemogram analysis. Fecal samples were subjected to fecal egg counts (Ueno and Gonçalves 1998).

### Abomasum collection and analyses of microscopic lesions and parasitic content

Abomasal samples were collected, and a portion from the fundic region was snap-frozen in liquid nitrogen and stored at -80 °C until RNA extraction. Another portion, including both fundic and pyloric regions, was fixed in 10% phosphate-buffered formalin (pH 7.2) and processed according to standard histological procedures.

Two to three full-thickness samples from fundic and pyloric regions were routinely processed for histopathological analysis. Sections stained with hematoxylin and eosin were evaluated by a pathologist under ‘blinded’ conditions. The evaluated parameters included lymphoid follicular hyperplasia and the number of lymphocytes/plasma cells, eosinophils, and neutrophils in the lamina propria. A cumulative histopathologic score was calculated as the sum of individual lesion scores. Additional sections were stained by blue toluidine to assess mast cells infiltration in lamina propria. Histological changes were graded using a semiquantitative scoring system (0 = normal, 1 = mild, 2 = moderate and 3 = severe lesions) based on the evaluation of ten fields at 400x magnification.

10% of the abomasal contents and mucosa were processed for parasite recovery, identification, and enumeration (Burden et al. 2024). *H. contortus* developmental stages were morphologically differentiated and quantified as follows: early L_4_ larvae, female L_4_ larvae, male L_4_ larvae, male L_5_ larvae, female L_5_ larvae, male adult worm and female adult worm (Ueno and Gonçalves 1998; Okino et al. 2026).

### RNA extraction and RT-qPCR for gene expression quantification

Total RNA was extracted from abomasal samples using QIAzol^®^ Lysis Reagent (Qiagen) and TissueRuptor (Qiagen), followed by purification on silica columns with the RNeasy Mini Kit (Qiagen). RNA concentration and purity were assessed by spectrophotometry (NanoDrop™ 2000, Thermo Scientific), and integrity was verified by 1.5% agarose gel electrophoresis through visualization of 28 S and 18 S ribosomal RNA bands. RNA (1800 ng) samples were treated with RQ1 RNase-Free DNase (Cat. M6101, Promega) and subsequently used for cDNA synthesis with the High-Capacity cDNA Reverse Transcription Kit (Applied Biosystems™, cat. 4368814) and oligo(dT) primers in a T100™ Thermal Cycler (Bio-Rad). All procedures were performed in accordance with the manufacturer’s instructions.

Real time quantitative PCR (qPCR) was performed using qPCRBIO SyGreen Mix (PCR Biosystems) on a QuantStudio 6 Pro (Thermofisher). Each reaction contained 20 ng of cDNA, 5 µL of 2X qPCRBIO SyGreen Mix, 100nM of ROX reference dye and 0.3 µM of each forward and reverse primer, in a final volume of 10 µL. Thermal cycling conditions consisted of an initial pre-incubation at 95 °C for 2 min, followed by 40 cycles of 95 °C for 15 s and 60 °C for 10 s. This was followed by melting curve analysis from 60 to 95 °C, with a ramp rate of 0.1 °C/s in the continuous acquisition mode. All samples were analyzed in duplicate, and no-template controls (NTC) and no-reverse transcription (RT-) controls were included in each run. Threshold values were manually set to a fixed level for each gene assay. Primer sequences and qPCR efficiencies were previously validated (Toscano et al. 2018, 2019, 2020; Okino et al. 2023). Gene expression levels were normalized using *GAPDH*, which was identified as the most stable reference gene among five candidates (*GAPDH*, *PPIA*,* YWHAZ*, *B2M* and *ACTB*). Relative gene expression was calculated as described by Livak and Schmittgen (2001). For each gene, the sample with the lowest expression level (highest ΔCq) was adopted as a calibrator. Twenty target genes involved in different pathways associated with the response to *H. contortus* infection were analyzed: alarmins (*IL33*), pro-inflammatory cytokines (*IL1B* and *TNFA*), inflammatory signaling (*NFKBIA*), anti-inflammatory cytokines (*TGFB* and *IL10*), Th2 cytokines (*IL5*, *IL13*, and *IL4*), complement molecules (*CFI* and *C7*), toll-like receptors (*TLR2*,* TLR4*, and *TLR7*), lectin receptors (*GAL11* and *GAL14*), IgE receptor (*MS4A2*) and mucin-related gene (*CLCA1*). Samples with no detectable amplification were assigned a Cq value of 40 (total number of cycles) for relative expression analysis, whereas gene assays with more than 30% of samples showing no amplification were excluded from the analysis.

### Statistical analysis

Statistical analyses were conducted using R software (R Core Team version 2026.01.1). Transformations were applied only when necessary, following a systematic assessment of normality and homoscedasticity. Hematological parameters were initially transformed using the Box-Cox method (PCV, erythrocytes, hemoglobin, lymphocytes, hematocrit by complete hemogram and neutrophils). When normality was not achieved, the orderNorm transformation was applied (leukocytes, MCV, MCH, eosinophils, monocytes and platelets). MCHC showed normal and homoscedastic distribution and was therefore not transformed. FEC data were normalized using Box-Cox transformation. Fold-change values of relative gene expression were normalized using the orderNorm transformation. Worm count data were transformed using the Yeo-Johnson method (bestNormalize package; Peterson 2021), except for female adult and male adult counts which presented normal and homoscedastic distributions. Transformed variables are indicated in the graphs by the prefix “t” preceding the parameter name. Hematological parameters, PCV, FEC, parasite counts and gene expression data were analyzed using analysis of variance (ANOVA), with animals treated as the within-subjects factor and breed, paddock, sex and sampling interval as between-subject factors (except for parasite counts and gene expression, which involved a single sampling interval and sex). ANOVA was performed using the anova_test(.) function from the rstatix package (Kassambara 2023). Post hoc comparisons (Tukey’s test) and residual diagnostics were conducted using the ea2(.) function (design = 7) from the easyanova package (Arnhold 2013). Pearson correlation coefficients (ρ) were estimated using metan package (Olivoto and Lúcio 2020) to assess associations between phenotypic traits and gene expression levels measured at the time of euthanasia. The scores of microscopic lesions were analyzed using the Kruskal-Wallis test followed by Dunn’s post hoc test with Bonferroni correction. The significance level was set at *p* < 0.05.

## Results

### Hematological analysis and fecal egg counts

**Fig. 1:**
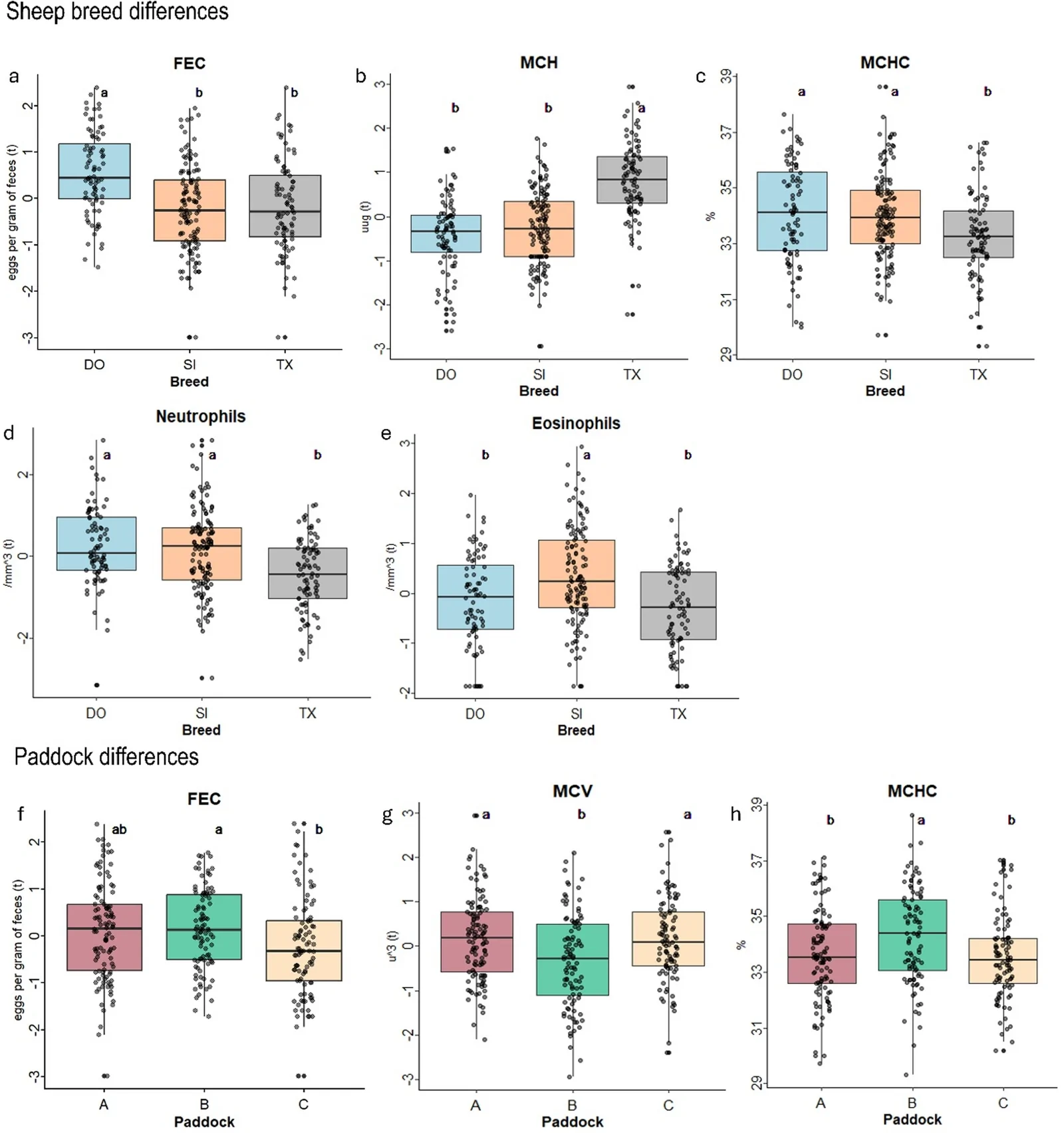
Complete hemogram results presenting significant interaction between breed and paddock. Mean values of packed cell volume (microhemocrit) (**a**), hematocrit (complete hemogram) (**b**), hemoglobin (**c**), erythrocytes (**d**) and platelets (**e**) among sheep breeds (White Dorper – DO – blue bars, Santa Inês – SI – orange bars and Texel – TX – grey bars) distributed into **A**, **B** and **C** paddocks. Different lowercase letters among sheep breeds indicated significant differences by Tukey test (*p* ≤ 0.05), while uppercase letters among paddocks indicated significant differences by Tukey test (*p* ≤ 0.05). The “t” letter in the y-axis title indicated transformed data

**Fig. 2:**
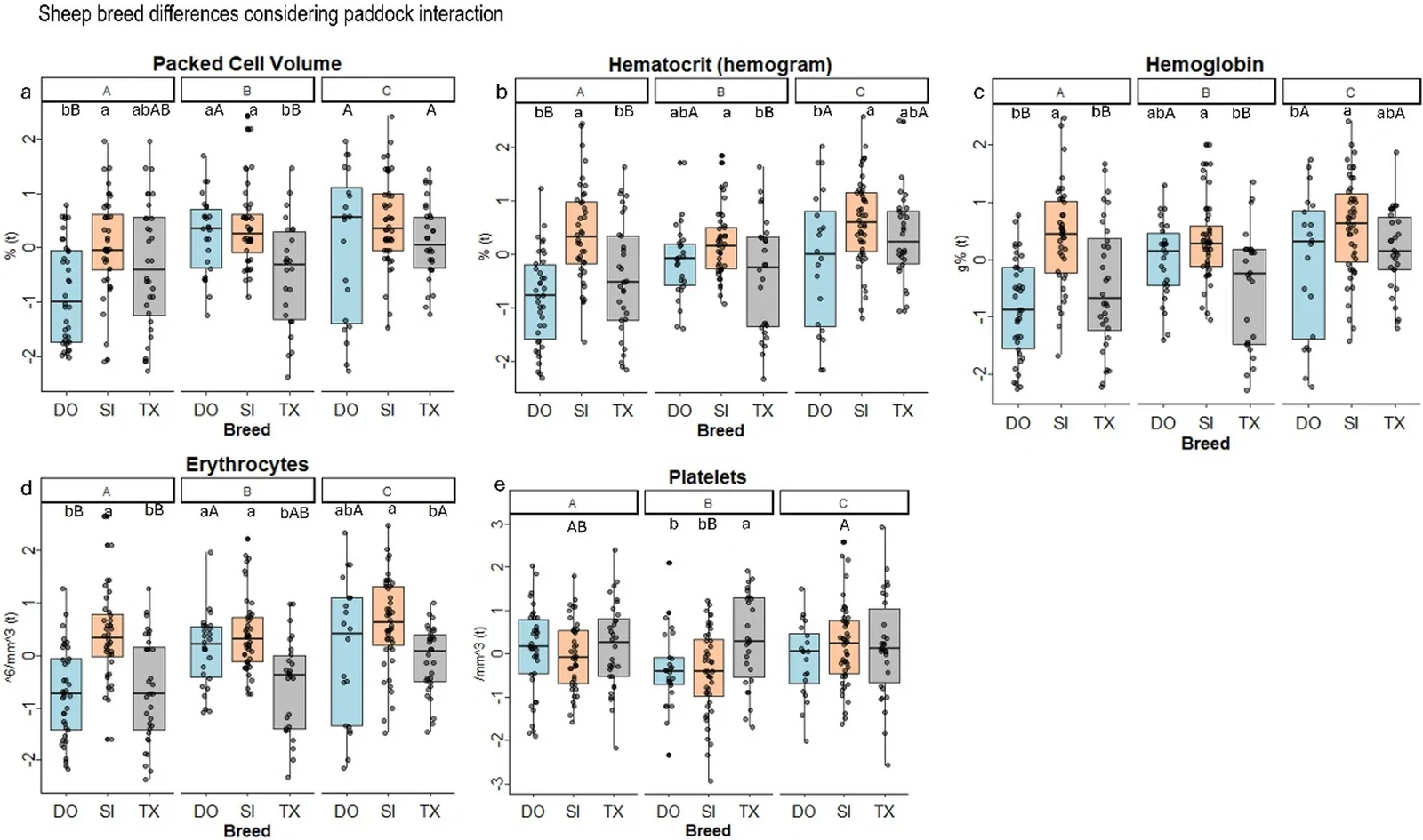
Complete hemogram results presenting significant interactions between breed and interval, including: leukocytes (**a**), lymphocytes (**b**) and monocytes (**c**) among sheep breeds (White Dorper – DO – blue bars, Santa Inês – SI – orange bars and Texel – TX – grey bars) at 105 and 189 days of age; or presenting significant interactions between paddock and interval, including: leukocytes (**d**), monocytes (**e**) and neutrophils (**f**) (paddock **A** – pink bars, paddock **B** -green bars and paddock **C** - yellow bars). Different lowercase letters among sheep breeds or paddocks indicated significant differences by Tukey test (*p* ≤ 0.05), while uppercase letters among intervals indicated significant differences by Tukey test (*p* ≤ 0.05). The “t” letter in the y-axis title indicated transformed data

Significant interaction effects between breed and paddock were detected for most red blood cells parameters, including PCV, erythrocytes, hemoglobin, hematocrit (hemogram) and platelets (Fig. 1). No significant interaction effects between sampling interval (D105 and D189) and either breed or paddock (A = high, B = intermediate, C = low *H. contortus* challenge) were observed for FEC or for any red blood cell parameters. In contrast, for the white blood cell parameters, significant interactions between sampling interval and breed were observed for leukocytes, lymphocytes and monocytes (Fig. 2a-c), as well as between sampling interval and paddock for leukocytes, monocytes and neutrophils (Fig. 2d-f). Significant interactions involving sex were also detected for PCV and MCV (Supplementary information 2).

**Fig. 3:**
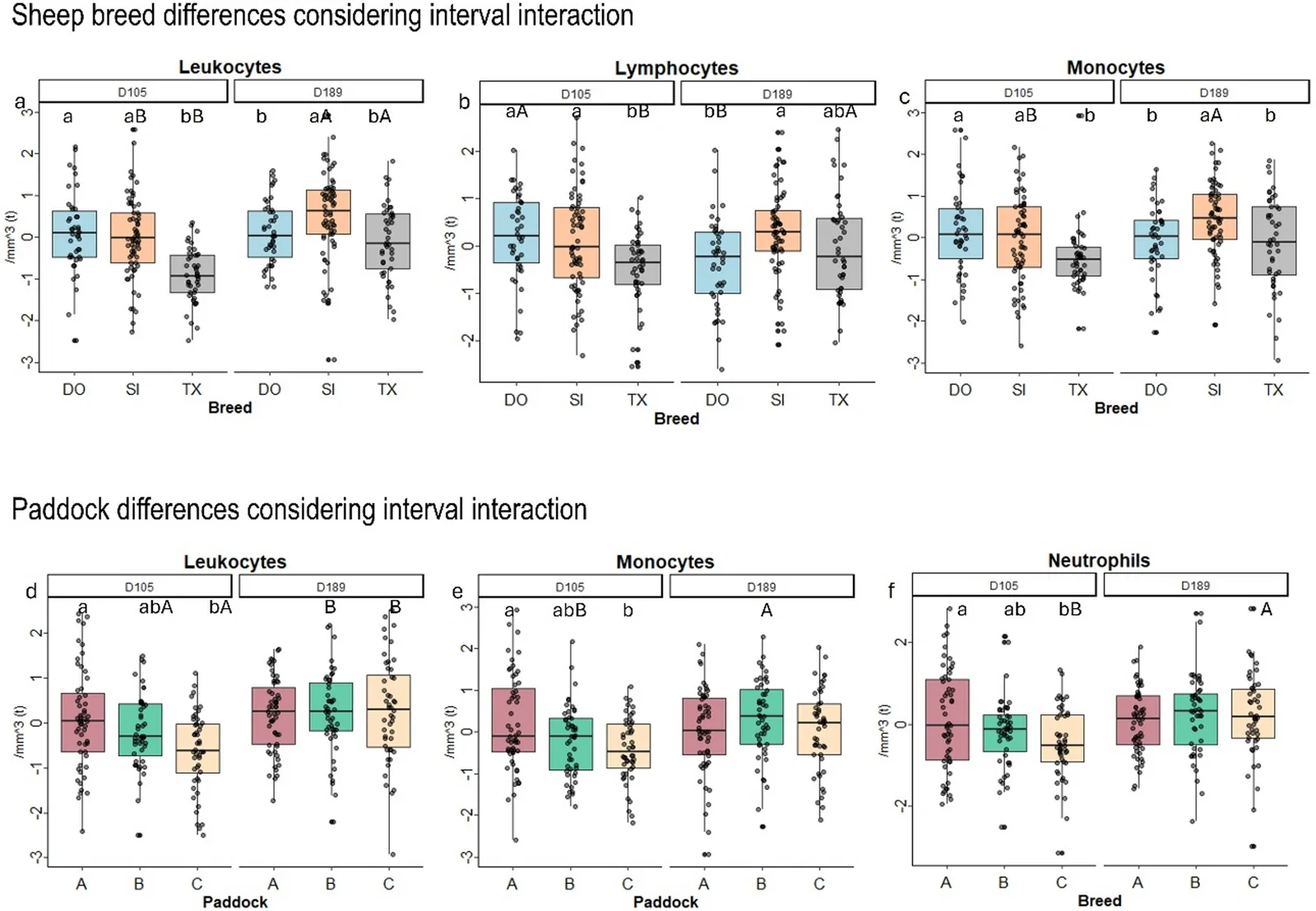
Sheep breed differences of mean values of fecal egg counts (**a**), MCH (mean corpuscular hemoglobin) (**b**), MCHC (mean corpuscular hemoglobin concentration) (**c**), neutrophils (**d**) and eosinophils (**e**) (White Dorper- DO – blue bars, Santa Inês – SI – orange bars and Texel – TX – grey bars); and differences among paddocks of FEC (**f**), MCV (mean corpuscular volume) (**g**) and MCHC (**h**) (**A** – pink bars, **B** – green bars and **C** – yellow bars). Different lowercase letters among sheep breeds indicate significant differences by Tukey test (*p* ≤ 0.05). The (t) in the y-axys title indicate transformed data

The FEC means were significantly higher in White Dorper lambs compared to Santa Inês (*p* < 0.001) and Texel (*p* < 0.001) (Fig. 3a). Lambs from the paddock B presented significantly higher FEC levels than those from paddock C (*p* = 0.029), whereas paddock A presented intermediate values that did not differ significantly from either B or C (Fig. 3f). No significant differences were observed between sampling intervals.

Mean corpuscular volume (MCV) was significantly higher in Texel compared to other breeds in both males (*p* < 0.001) and females (*p* < 0.001). In contrast, White Dorper males exhibited significantly lower MCV values (*p* = 0.034) than Santa Inês males (Supplementary information 2b). Accordingly, mean corpuscular hemoglobin (MCH) was also significantly higher (*p* < 0.001), whereas mean concentration of hemoglobin corpuscular (MCHC) was significantly lower in Texel compared to White Dorper (*p* = 0.001) and Santa Inês (*p* = 0.002) (Fig. 1b and c). Regarding comparison among the paddocks, paddock B presented significantly lower MCV (*p* < 0.001) and higher MCHC values compared to paddock A (*p* = 0.019) and C (*p* = 0.013) (Fig. 1g and h). Additionally, significantly higher MCH (*p* < 0.001) and MCHC (*p* < 0.001) values were observed at D189 compared to D105 (Supplementary information 1c and 1d).

Regarding the red blood cell parameters, hematocrit (from complete hemogram) (*p* < 0.001), hemoglobin concentration (*p* < 0.001) and erythrocyte counts (*p* < 0.001) were significantly higher in Santa Inês compared to other breeds, but only in paddock A (Figs. 2b-d). Differences among breeds were milder in paddock B, with no significant differences between White Dorper and Santa Inês, and were minimal in paddock C (Fig. 2). Among the red blood cell parameters used to assess anemia, PCV measured by microhematocrit method showed the smallest differences among breeds (Fig. 2a). When comparing paddocks within breeds, Santa Inês lambs maintained consistent red blood cell parameters (PCV, hematocrit, hemoglobin and erythrocytes) across all paddocks. In contrast, White Dorper lambs showed the lowest values in paddock A compared to B and C, whereas Texel lambs exhibited the highest values in paddock C and generally the lowest values in paddock A. No significant differences between sampling intervals were observed for hemoglobin concentration or PCV. However, erythrocytes counts and hematocrit (from complete hemogram) were significantly higher at D105 compared to D189 (*p* = 0.012 and *p* = 0.014, respectively) (Supplementary information 1a and 1b).

Platelets levels differed significantly among breeds only in the paddock B, where Texel exhibited the highest values (*p* < 0.001) (Fig. 2e). No significant breed differences were observed in paddocks A and C. Within breeds, Santa Inês lambs in paddock C showed significantly higher platelet counts than those in paddock B (*p* < 0.001), with intermediated values observed in paddock A (Fig. 2e).

Regarding white blood cell parameters, Texel lambs showed significantly lower levels of neutrophils (*p* < 0.001) (Fig. 3d), total leukocytes at D105 (*p* < 0.001) (Fig. 2a), lymphocytes at D105 (*p* = 0.01) (Fig. 2b) and monocytes at D105 (*p* < 0.05) (Fig. 2c) compared to other breeds. Eosinophil counts were significantly higher in Santa Inês lambs (*p* < 0.001) (Fig. 3e). At D189, total leukocytes (*p* < 0.05), lymphocytes (Santa Inês vs. White Dorper, *p* = 0.001) and monocytes (*p* < 0.005) were also significantly higher in Santa Inês compared to other breeds, however, lymphocyte levels did not differ between Santa Inês and Texel (Fig. 3).

Regarding paddock effects, paddock C showed significantly lower total leukocyte (*p* < 0.001) and monocyte (*p* = 0.005) counts compared to paddock A at D105, with intermediate values observed in paddock B (Fig. 3d and e). Additionally, eosinophil (*p* < 0.001) and neutrophil (*p* = 0.001) counts were significantly higher at D189 compared to D105 (Supplementary information 1).

### Worm counts in abomasum

A significant interaction between sheep breed and paddock was observed only for L_5_ female worm counts, while no significant interactions were detected for other parasitic stages.

**Fig. 4:**
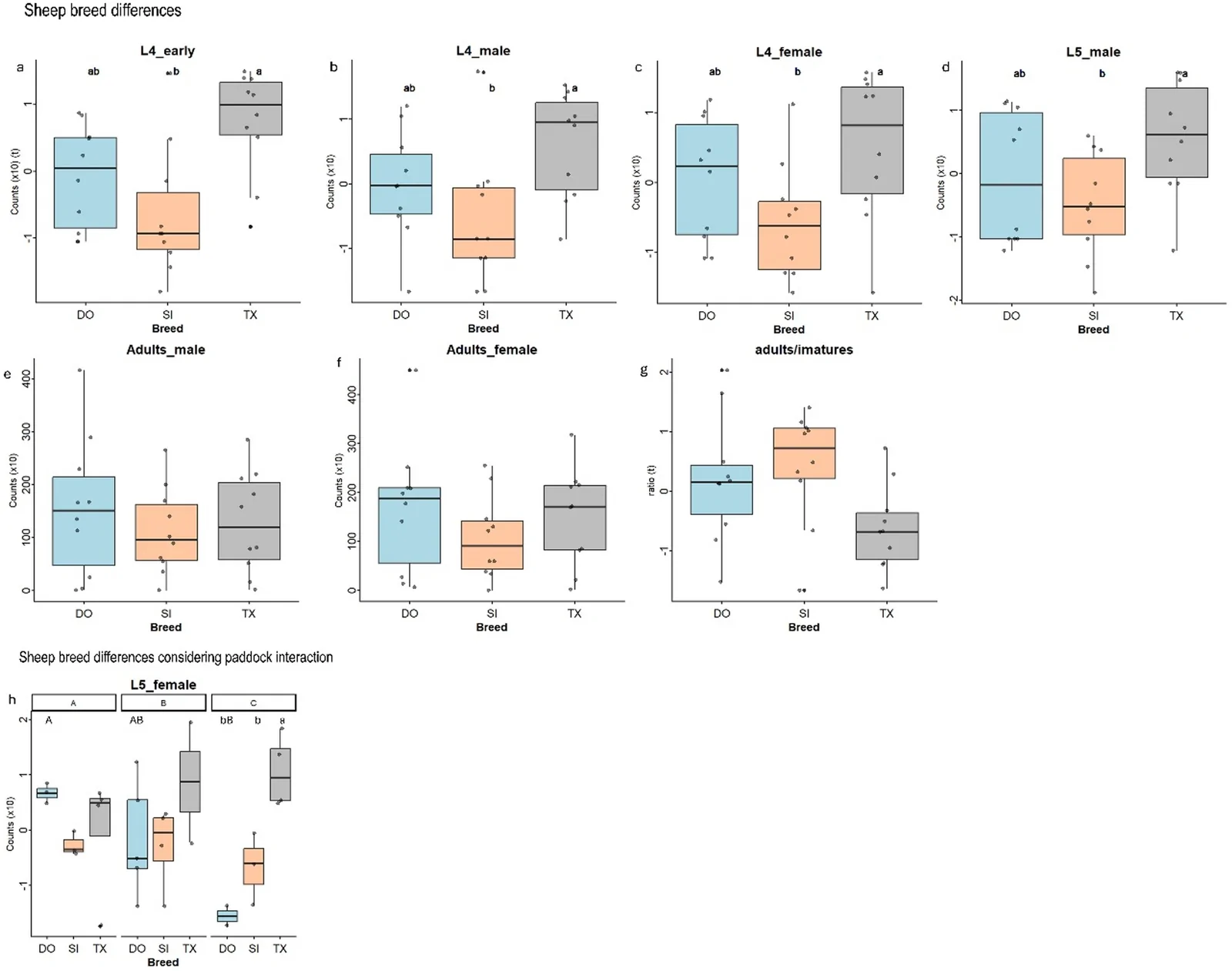
Parasitic stage counts in the abomasum among sheep breeds, including (**a**) early L_4_ larvae, (**b**) male L_4_ larvae, (**c**) female L_4_ larvae, (**d**) male L_5_ larvae, (**e**) male adults, (**f**) female adults and (**g**) adult/immature worm ratio; and among sheep breeds presenting significant interaction with paddocks (**A**, **B** and **C**) for (**h**) female L_5_ larvae; in lambs (White Dorper – DO – blue bars, Santa Inês – SI – orange bars and Texel – TX – grey bars) of approximately 210 days of age naturally infected with *H. contortus*. Different lowercase letters among sheep breeds indicated significant differences by Tukey test (*p* ≤ 0.05), while uppercase letters among paddocks indicated significant differences by Tukey test (*p* ≤ 0.05). The “t” letter in the y-axis title indicated transformed data

Significant differences in the worm counts among breeds were observed from early L_4_ to L_5_ stages, for both male and female parasites. Across these stages, Texel lambs exhibited significantly higher counts compared to Santa Inês (early L_4_, *p* = 0.001; male L_4_, *p* = 0.019; female L_4_, *p* = 0.042; male L_5_, *p* = 0.025; female L_5_, *p* = 0.034). White Dorper presented intermediate values, with no significant differences compared to either breed. For L_5_ female larvae counts, no differences among the paddocks were observed for Santa Inês and Texel. In contrast, White Dorper lambs in paddock C had significantly lower counts compared than those in paddock A (*p* = 0.022), with intermediate values observed in paddock B. Adult worm counts (males and females) did not differ among breeds (Fig. 4).

### Relative quantification of gene expression and correlations with parasitic stages counts

**Fig. 5:**
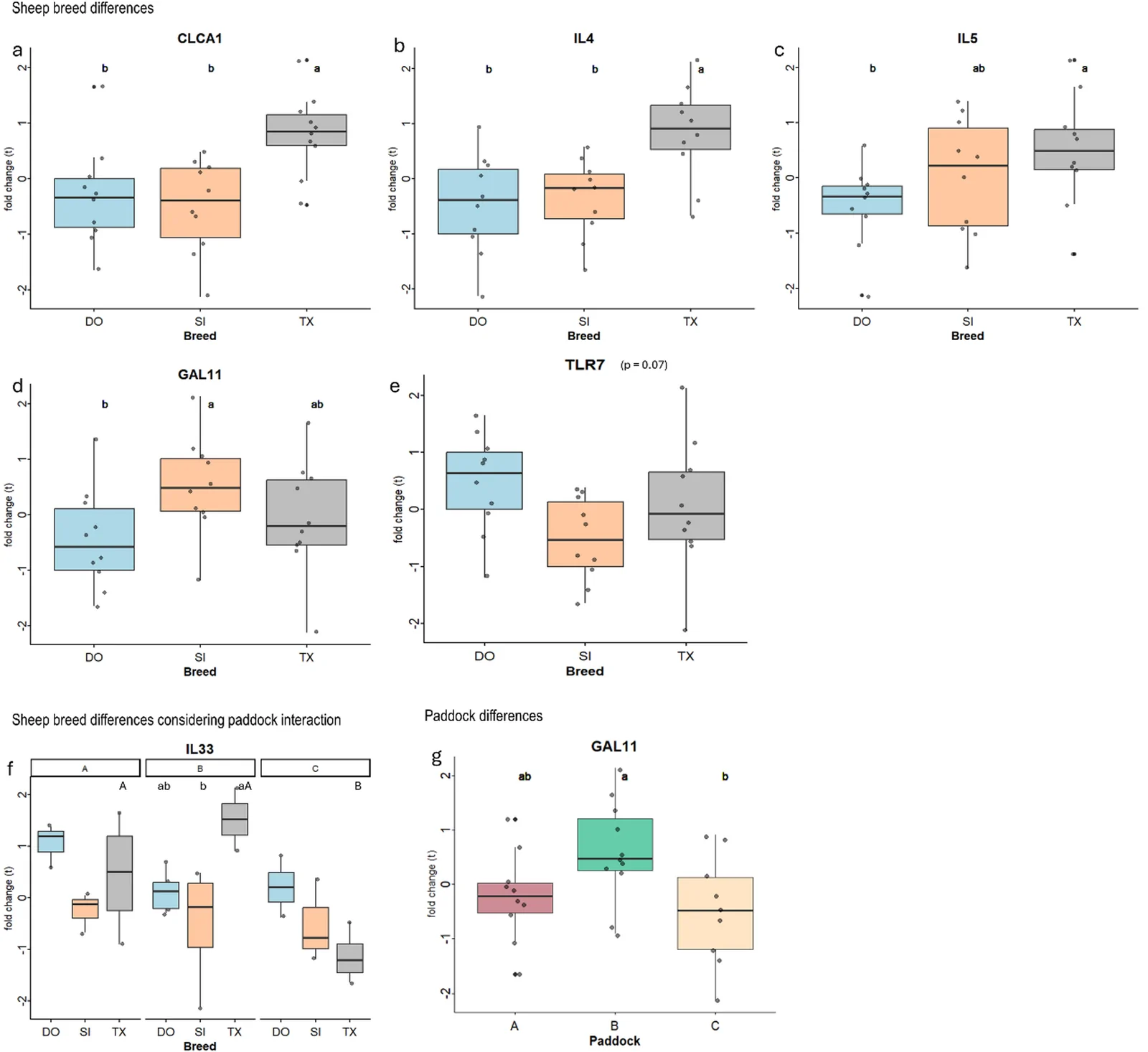
Relative quantification of gene expression in the fundic abomasum among different breeds, including: (**a**) *CLCA1*, (**b**) *IL4*, (**c**) *IL5*, (**d**) *GAL11*, (**e**) *TLR7*; among different sheep (White Dorper – DO – blue bars, Santa Inês – SI – orange bars and Texel – TX – grey bars) presenting significant interaction with paddocks (**A**, **B** and **C**) for (**f**) *IL33*; or among different paddocks for (**g**) *GAL11*, of lambs naturally infected with *H. contortus*. Different letters among the sheep breeds or paddock indicated significant differences by Tukey test (*p* < 0.05), while uppercase letters among paddocks indicated significant differences by Tukey test (p ≤ 0.05). The ”t” letter in the y-axis title indicated transformed data

Relative gene expression comparing sheep breeds are presented in Fig. 5 and Supplementary information 3. A significant interaction between breed and paddock was observed only for *IL33* transcripts.

Texel lambs exhibited significantly higher expression levels of *CLCA1* (*p* < 0.05) and *IL4* (*p* < 0.01) compared to other breeds, while *IL5* expression was higher in Texel only in comparison with White Dorper (*p* = 0.028).

Santa Inês lambs showed significantly higher *GAL11* expression (*p* = 0.042) and a trend toward lower *TLR7* expression (*p* = 0.07) compared to White Dorper.

Regarding paddock effects, paddock B exhibited significantly higher *GAL11* mRNA levels than paddock C (*p* = 0.021), with paddock A showing intermediate levels.

Significant differences in *IL33* expression among breeds were observed only in paddock B, where Texel showed higher expression than Santa Inês (*p* = 0.018) and a trend toward higher expression compared to White Dorper (*p* = 0.10). No significant differences among paddocks were observed for Santa Inês and White Dorper. In contrast, Texel showed significantly lower *IL33* expression in paddock C compared to paddocks A (*p* = 0.025) and B (*p* = 0.002).

No significant differences among breeds were detected for *TGF*,* TLR2*,* NFKBIA*,* IFNG*,* IKBKB*,* IL33*,* C7*,* CFI*,* GAL14* and *TLR4* (Supplementary information 3). Expression levels of *IL13*,* IL1β*,* IL10*,* TNFA* and *MS4A2* genes were low, with several samples showing no amplification, therefore, these genes were excluded from further analysis.

Correlations between gene expression and parasite stage counts with coefficients lower than − 0.3 (pink squares) or higher than 0.3 (blue squares) are presented in Table 2

**Table 2:**
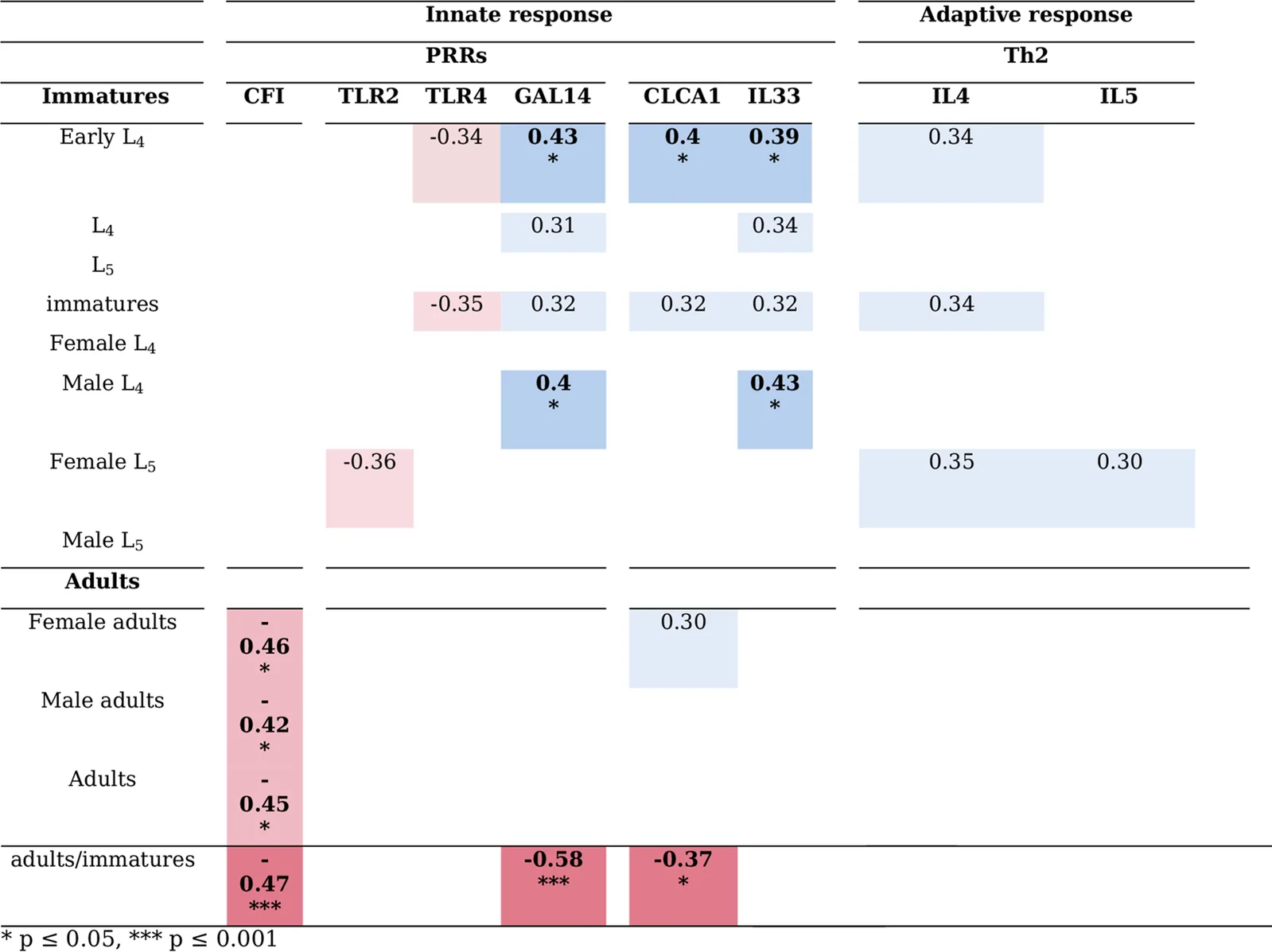
Correlation by Pearson test among parasitic stage counts (early L_4_, male L_4_, female L_4_, L_4_, male L_5_, female L_5_, L_5_, immatures, male adult, female adult, adult, total worm, adult/immature ratio) and gene expression levels (*CFI*,* TLR2*,* TLR4*,* GAL14*,* CLCA1*,* IL33*,* IL4* and *IL5*)

Significant negative correlations were observed between all adult worm counts and *CFI* transcript levels. Early L_4_ and female L_5_ counts showed trends toward negative correlations with *TLR4* and *TLR2* mRNA levels, respectively.

Early L_4_ counts were positively correlated with *GAL14*, *CLCA1* and *IL33.* Weaker positive correlations were observed for the later parasite stages (L_4_ and L_5_ counts). Male L_4_ counts were also positively correlated with *GAL14* and *IL33*. Additionally, larval counts showed trends toward positive correlations with Th2 cytokine transcripts (*IL4* and *IL5*).

The ratio adults per immatures was negatively correlated with *CFI*, *GAL14* and *CLCA1* mRNA levels.

### Microscopic lesions in the abomasum

Significantly higher scores of lymphocytes and plasm cells in the lamina propria of both abomasal fundic (*p* = 0.02) and pyloric (*p* < 0.001) regions of the abomasum were observed in Texel compared to the other breeds.

Neutrophil and mast cell scores in the lamina propria were also significantly higher in Texel compared to White Dorper in both the fundic (*p* < 0.001 and *p* = 0.049, respectively) and pyloric regions (*p* = 0.008 and *p* = 0.004, respectively), whereas Santa Inês showed intermediate scores.

Lymphoid follicular hyperplasia scores were significantly higher in Santa Inês compared to White Dorper in the fundic region (*p* = 0.03), with Texel showing intermediate values. In contrast, in the pyloric region, Texel exhibited higher scores than White Dorper (*p* = 0.02), while Santa Inês again showed intermediated values.

No significant differences among breeds were observed for eosinophils scores.

Regarding the cumulative histopathological scores, Texel showed higher values than White Dorper in both fundic and pyloric regions, whereas differences between Texel and Santa Inês were observed only in the pyloric region (Table 3). No significant differences were detected among paddocks.

**Table 3:** Histopathologic findings in fundic and pyloric regions of the abomasum from lambs of different sheep breeds (White Dorper, Santa Inês and Texel) naturally infected with *Haemonchus contortus*

Fundic region	Lymphocytes and plasma cells	Eosinophils	Neutrophils	Lymphoid follicular hyperplasia	Cumulative score	Mast cells
White Dorper	1a	0	0a	0a	1a	0.5a
Santa Inês	1a	0	0ab	1b	2ab	1ab
Texel	2b	0	1b	1ab	3.5b	1b
*Pyloric region*						
White Dorper	2a	0	0a	1a	3a	1a
Santa Inês	2a	0	0.5ab	1ab	3.5a	1ab
Texel	3b	0	1b	2b	6b	1b

Different letters among the sheep breeds indicated significant differences by Kruskal Wallis test followed by Bonferroni’s multiple comparison (*p* < 0.05)

## Discussion

Santa Inês lambs exhibited enhanced resistance to *H. contortus* infection compared to Texel and White Dorper, as indicated by lower FEC values, higher red blood cell parameters (PCV, hematocrit, hemoglobin and erythrocytes), and lower larval counts in the abomasum. Significant differences were observed among paddocks. Lambs raised in paddock C (low parasitic challenge) showed a more favorable combination of phenotypic health parameters, including lower FEC and higher red blood cell values, particularly in Texel and White Dorper breeds. All the red blood cell parameters showed significant breed x paddock interactions.

Notably, Santa Inês lambs remained unaffected by the paddock conditions, suggesting a robust and well-regulated response capable of maintaining homeostasis across varying levels of parasitic challenge. In contrast, Texel and White Dorper lambs exhibited marked variation in response to different paddock conditions. In Texel lambs, hematocrit and hemoglobin were significantly higher in paddock C compared to paddocks A and B, while erythrocytes were higher in paddock C than in paddock A, with intermediate values observed in paddock B. Similarly, White Dorper lambs raised in paddock C exhibited improved red blood cell parameters compared to those in paddocks A and B.

At D105, which may represent the acute phase of haemonchosis, lambs in paddock C showed significantly lower circulating monocyte levels than those in paddock A, with intermediate values observed in paddock B. Total leukocyte levels followed a similar pattern. Monocyte trafficking from the bloodstream to peripheral tissues is a hallmark of acute infection and inflammation (Shi and Pamer 2011), supporting the higher circulating levels observed in lambs from paddock A (high parasitic challenge).

Expression of *IL33* also varied among paddocks in Texel lambs, with significantly lower levels detected in paddock C. This likely reflects reduced parasite burden and, consequently, lower host inflammatory responses. *IL33* plays a key role in the early phase of GIN infections, acting as an alarmin released by damaged epithelial cells that activates type 2 innate lymphoid cells (*ILC2*) and promotes Th2 polarization (Kondo et al. 2008; Yasuda et al. 2012).

The L_5_ female larvae counts in White Dorper lambs were significantly lower in paddock C compared to paddock A, with intermediate values in paddock B. These findings indicate that lambs differ in their ability to cope with environmental *H. contortus* challenge loads according to breed.

The higher MCV, combined with lower erythrocyte counts observed in Texel lambs compared to the other breeds, indicates macrocytic anemia, likely associated with enhanced erythroblasts regeneration following blood loss induced by haemonchosis (Jiménez-Penago et al. 2021). Regenerative macrocytic anemia results from erythropoietin stimulation of the bone marrow, leading to increased production of erythroblasts and reticulocytes. Since reticulocytes have a larger volume than mature erythrocytes, their increased proportion elevates the average red blood cell volume, thereby increasing MCV (Jiménez-Penago et al. 2021). The elevated MCV observed in Texel likely contributes to the higher MCH and lower MCHC also detected in this breed. This may also explain why the hematocrit and hemoglobin means were similar between Texel and the more resistant Santa Inês breed, despite lower erythrocyte counts in Texel under low parasitic challenge conditions (paddock C). Overall, these findings suggest a distinct regulation of erythropoiesis in Texel compared to other breeds.

In our previous study, increased mRNA expression of *TLR2* and *TLR4* was negatively correlated to the L_4_ larval counts (Okino et al. 2025). In the present study, similar negative correlations were observed, although they did not reach statistical significance.

In mice deficient in *TLR2* and *TLR4*, increased susceptibility to *Ascaris summ* infection has been reported and was associated with reduced eosinophil levels and decreased production of secretory IgA antibodies in pulmonary mucosa (Nogueira et al. 2021). These findings suggest that *TLR2* and *TLR4* signaling contributes to the induction of eosinophilic responses and mucosal IgA production. Toll-like receptors such as *TLR2* and *TLR4* recognize a wide range of pathogen associated molecular patterns, including those from extracellular pathogens as well as intracellular pathogens within endosomes and lysosomes. Beyond their role as innate immune sensor, they also act as key modulators linking innate and adaptive immune responses (Mukherjee et al. 2016). In line with these observations, *TLR2* expression was significantly higher in Santa Inês compared to Texel in our previous study, with a similar trend observed for *TLR4* transcripts (Okino et al. 2025). On other hand, in the present study, a similar pattern was observed for *TLR4* transcripts, but not for *TLR2*. This divergence of results may be explained by the absence of β-globin A allele in the experimental animals of the present study, in contrast to our previous study, in which Santa Inês group comprised 3/8 BB, 4/8 AB and 1/8 AA genotypes (Okino et al. 2025). The presence of the A allele has been associated with increased resistance to *H. contortus* infection. In particular, AA homozygous lambs show higher *TLR2* mRNA expression and increased eosinophils recruitment in abomasal mucosa compared to animals with the BB haplotype, as demonstrated in Morada Nova lambs (Okino et al. 2023). Eosinophils have been shown to exert larvicidal activity against *H. contortus* under in vitro conditions, reducing parasite establishment in sheep (Terefe et al. 2007).

In the present experiment, increased peripheral eosinophilia was observed in Santa Inês compared to Texel and White Dorper lambs, however, no differences in abomasal eosinophil infiltration were detected among breeds.

A lack of differences in abomasal eosinophilia has also been reported, even in the presence of lower worm burden and higher blood eosinophil levels in Barbados Black Belly (resistant) compared to INRA-401 (susceptible) sheep primarily infected with *H. contortus* (Terefe et al. 2009).

*GAL11* is specifically induced in gastrointestinal epithelial cells following parasitic infection and is associated with increased mucus adhesiveness. This molecule has been suggested to play an important role in reducing *H. contortus* motility (Robinson et al. 2011). Furthermore, *GAL11* transcripts were upregulated in resistant genotype of Morada Nova lambs infected with *H. contortus* (Okino et al. 2023). Our results are consistent with these findings, as *GAL11* expression was significantly higher in Santa Inês compared to White Dorper, with intermediate expression observed in Texel.

Texel lambs exhibited significantly higher counts of all immature parasite stages compared to Santa Inês lambs, however, FEC levels and adult worm burdens were similar between these breeds. The L_5_ female counts differed significantly among breeds only under low parasitic challenge, with Texel showing the highest values.

Regarding leukocyte profiles, Texel lambs showed significantly lower neutrophil counts, whereas Santa Inês lambs exhibited significantly higher eosinophil levels compared to the other breeds. At D105, Texel had significantly lower lymphocyte and monocyte counts, while at D189, these cell populations were significantly higher in Santa Inês. *CLCA1* has been associated with mucus dynamics, particularly in facilitating mucus processing and clearance (Nyström et al. 2018), and has been reported to be upregulated in resistant Morada Nova lambs challenged with *H. contortus* (Okino et al. 2023). In the present study, Texel lambs showed significantly higher *CLCA1* expression, along with increased expression of Th2-related cytokines, including *IL4* and *IL5* compared to the other breeds.

Although Texel lambs exhibited increased activation of Th2-related genes, this was not associated with improved parasite control. This finding suggests that mucus-related responses and Th2-type immunity may have been overstimulated as a consequence of higher burden of immature parasites in this breed. Consistent with this interpretation, most of microscopic lesion’s parameters were also significantly elevated in Texel compared to other breeds, supporting the gene expression and parasite count results.

Regarding the correlation between parasite stage counts and immune-related transcripts, the role of *CFI* in later stages of *H. contortus* infection was reinforced in the present study, as similar findings were observed in our previous experiment using a different group of animals (Okino et al. 2025). *CFI* has a major role in the control of all complement pathways, through degradation of activated *C3b* and *C4b* in association with cofactors such as factor H, C4b-binding protein, complement receptor 1 (Nilsson et al. 2011). In our previous (Okino et al. 2025) and current studies comparing Santa Inês, White Dorper and Texel breeds, no significant differences in *CFI* transcript levels were detected among breeds. However, increased expression of this complement-related gene has been reported in resistant Canary Islands sheep following primary infection with *H. contortus*, but not in susceptible animals (Guo et al. 2016). Differences in necropsy timing may explain this discrepancy. In the study by Guo et al. (2016), necropsy was performed during acute phase of infection (20 days after infection), whereas in our studies, necropsies were conducted during chronic phase.

Our results demonstrate that resistance to *H. contortus* infection is both breed-dependent and modulated by the level of environmental challenge. The Santa Inês breed exhibited consistent resistance, characterized by stable red blood cell parameters, lower parasite burdens, higher peripheral eosinophilia and lymphocytosis, and increased local expression of *GAL11*, regardless of challenge intensity. In contrast, Texel and White Dorper showed greater susceptibility, with responses varying according to infection intensity and associated with increased physiological impairment. Notably, the enhanced activation of genes associated with Th2-type immune responses and mucus dynamics in Texel did not translate into improved parasite control, indicating that a more intense immune response is not necessarily more effective.

These findings highlight the importance of both breed-specific traits and environmental interactions in shaping resistance to parasitic infections. They also underscore the potential of adapted breeds, such as Santa Inês, for sustainable parasite control strategies, while emphasizing the need to account for within-breed variability when designing genetic improvement programs.

## Conclusion

The naturally resistant Santa Inês breed maintained consistent phenotypic characteristics across different levels of parasitic challenge, whereas White Dorper and Texel breeds exhibited increased impact of parasitism under moderate to high challenge conditions. Notably, under low challenge, Texel showed reduced resistance to haemonchosis compared to White Dorper, as evidenced by lower erythrocyte counts and higher burdens of immature parasites. Higher levels of circulating eosinophils and lymphocytes, along with increased local expression of *GAL11*, were observed in Santa Inês compared to other breeds, and may represent key host mechanisms contributing to improved control of the infection.

## Supplementary Information

Below is the link to the electronic supplementary material.

Supplementary Material 1

Supplementary Material 2

Supplementary Material 3

Supplementary Material 4

## Data Availability

All data were inserted as raw data file, other information was included as supplementary information 1 to 3. Additional information related to this study will be available if requested.
