# Supplementary material for: Sheep breed-specific response to environment challenge against Haemonchus contortus and effect on immuno-hematological parameters
Source: Vet Res Commun. 2026 Jun 6;50(5):372. doi: 10.1007/s11259-026-11304-2 (PMC13242422; doi:10.1007/s11259-026-11304-2)

Supplementary information 1. Complete hemogram results presenting significant differences between intervals. Mean values of hematocrit (by complete hemogram) (a), erythrocytes (b), MCH (mean corpuscular hemoglobin) (c), MCHC (mean corpuscular hemoglobin concentration) (d), eosinophils (e), neutrophils (f) and platelets (g) of experimental lambs at 105 (pink bars) and 189 (green bars) days of age. Different lowercase letters among intervals indicated significant differences by Tukey test (p ≤ 0.05). The “t” letter in the y-axis title indicated transformed data.


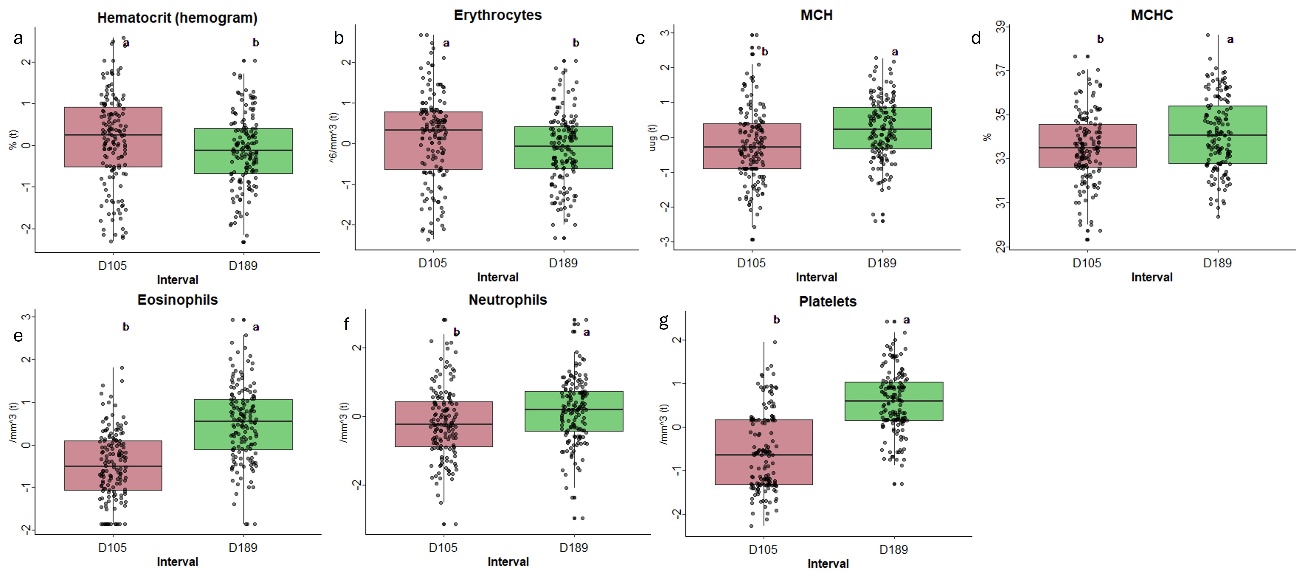

Supplement: Supplementary file 1 — Supplementary Material 1 [file 11259_2026_11304_MOESM1_ESM.docx]
