# Supplementary material for: Sheep breed-specific response to environment challenge against Haemonchus contortus and effect on immuno-hematological parameters
Source: Vet Res Commun. 2026 Jun 6;50(5):372. doi: 10.1007/s11259-026-11304-2 (PMC13242422; doi:10.1007/s11259-026-11304-2)

Supplementary information 2. Complete hemogram results presenting significant interaction between breed and sex, including mean values of hematocrit (by complete hemogram) (a) and MCV (mean corpuscular volume) (b) among lamb breeds(White Dorper – DO – blue bars, Santa Inês – SI – orange bars and Texel – TX – grey bars) distributed in females (F) and males (M) groups. And mean values of lymphocytes (c) by sex of experimental lambs (Females – F – pink bar and Males – M – blue bar). Different lowercase letters among intervals indicated significant differences by Tukey test (p ≤ 0.05). The “t” letter in the y-axis title indicated transformed data.


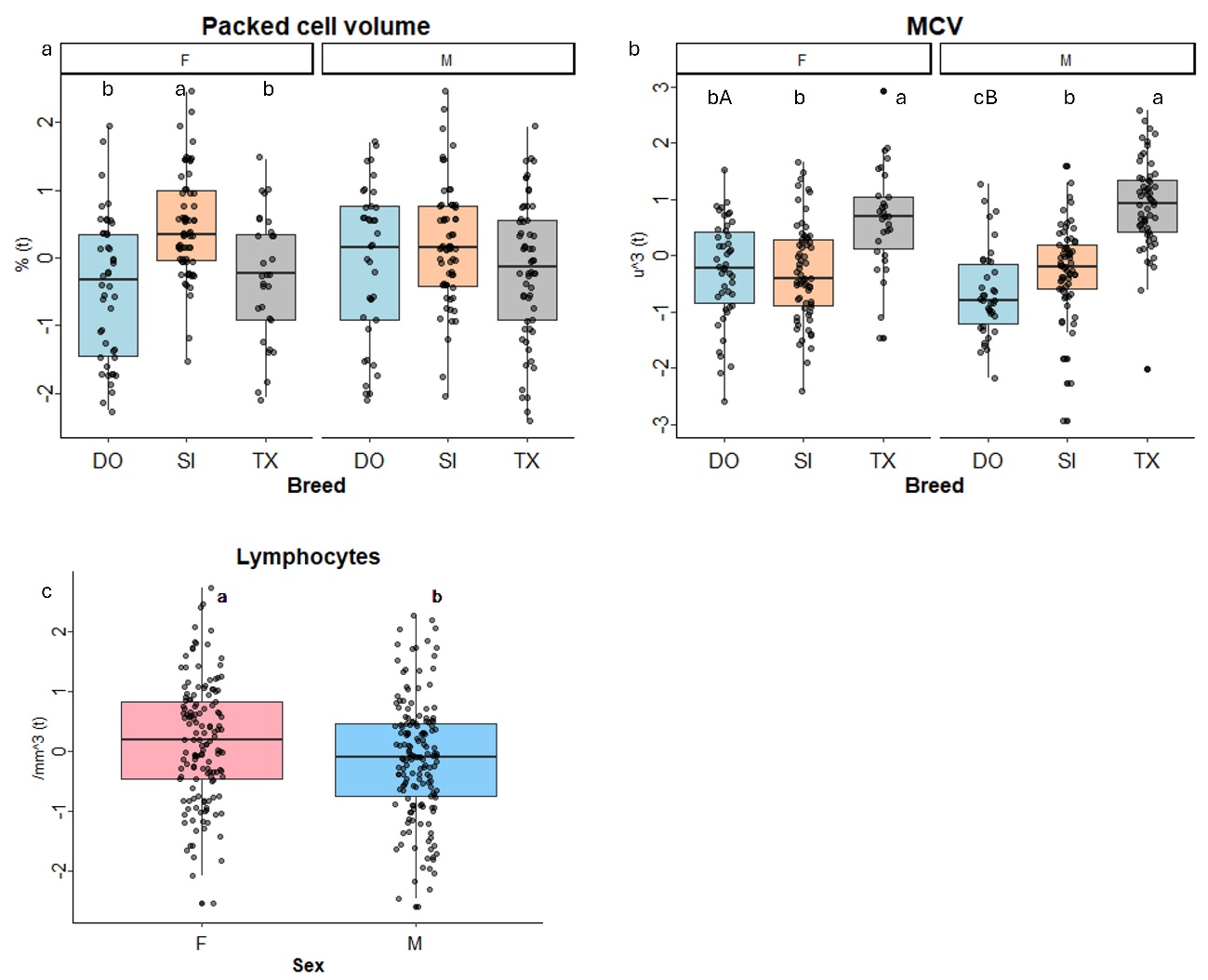

Supplement: Supplementary file 2 — Supplementary Material 2 [file 11259_2026_11304_MOESM2_ESM.docx]
