# Supplementary material for: Sheep breed-specific response to environment challenge against Haemonchus contortus and effect on immuno-hematological parameters
Source: Vet Res Commun. 2026 Jun 6;50(5):372. doi: 10.1007/s11259-026-11304-2 (PMC13242422; doi:10.1007/s11259-026-11304-2)
Supplement: Supplementary file 3 — Supplementary Material 3 [file 11259_2026_11304_MOESM3_ESM.docx]

Supplementary information 4. Histopathologic findings in fundic (F) and pyloric (P) region of the abomasum from lambs of different sheep breeds (White Dorper, Santa Inês and Texel) naturally infected with *Haemonchus contortus*.

| Breed | Animal | Lamina propria  lymphocytes and  plasma cells | | Lamina propria  eosinophils | | Lamina propria  neutrophils | | Lymphoid follicular  hyperplasia | | Cumulative  histopathologic score | | Lamina propria mast cells | |
| --- | --- | --- | --- | --- | --- | --- | --- | --- | --- | --- | --- | --- | --- |
|  |  | F | P | F | P | F | P | F | P | F | P | F | P |
| DO | 508 | 2 | 2 | 0 | 0 | 1 | 0 | 0 | 1 | 3 | 3 | 1 | 1 |
| DO | 512 | 1 | 2 | 0 | 0 | 0 | 1 | 0 | 1 | 1 | 4 | 1 | 1 |
| DO | 526 | 1 | 1 | 0 | 0 | 0 | 0 | 0 | 1 | 1 | 2 | 1 | 1 |
| DO | 527 | 1 | 2 | 0 | 0 | 0 | 0 | 0 | 1 | 1 | 3 | 0 | 0 |
| DO | 529 | 2 | 3 | 0 | 0 | 0 | 0 | 0 | 2 | 2 | 5 | 1 | 1 |
| DO | 533 | 1 | 1 | 0 | 0 | 0 | 0 | 0 | 1 | 1 | 2 | 0 | 0 |
| DO | 535 | 1 | 1 | 0 | 0 | 0 | 0 | 0 | 1 | 1 | 2 | 0 | 0 |
| DO | 542 | 1 | 2 | 0 | 0 | 0 | 0 | 0 | 1 | 1 | 3 | 0 | 0 |
| DO | 552 | 1 | 2 | 0 | 0 | 0 | 1 | 1 | 2 | 2 | 5 | 0 | 1 |
| DO | 554 | 2 | 2 | 0 | 0 | 0 | 0 | 2 | 1 | 4 | 3 | 1 | 1 |
| SI | 803 | 1 | 1 | 0 | 0 | 0 | 0 | 1 | 1 | 2 | 2 | 0 | 0 |
| SI | 826 | 1 | 2 | 0 | 0 | 0 | 1 | 1 | 2 | 2 | 5 | 1 | 1 |
| SI | 827 | 1 | 2 | 0 | 0 | 0 | 1 | 1 | 1 | 2 | 4 | 1 | 1 |
| SI | 834 | 1 | 2 | 0 | 0 | 1 | 1 | 1 | 2 | 3 | 5 | 1 | 1 |
| SI | 844 | 1 | 1 | 0 | 0 | 0 | 0 | 1 | 1 | 2 | 2 | 0 | 0 |
| SI | 845 | 1 | 1 | 0 | 0 | 0 | 0 | 1 | 1 | 2 | 2 | 0 | 0 |
| SI | 849 | 1 | 1 | 0 | 0 | 1 | 1 | 1 | 1 | 3 | 3 | 0 | 0 |
| SI | 857 | 2 | 2 | 0 | 0 | 0 | 0 | 1 | 2 | 3 | 4 | 2 | 2 |
| SI | 867 | 2 | 2 | 0 | 0 | 0 | 0 | 0 | 1 | 2 | 3 | 2 | 2 |
| SI | 873 | 2 | 2 | 0 | 0 | 1 | 1 | 1 | 2 | 4 | 5 | 2 | 2 |
| TX | 545 | 2 | 3 | 0 | 0 | 0 | 1 | 0 | 2 | 2 | 6 | 1 | 1 |
| TX | 555 | 2 | 2 | 0 | 0 | 1 | 1 | 1 | 2 | 4 | 5 | 2 | 2 |
| TX | 565 | 2 | 3 | 2 | 2 | 0 | 1 | 0 | 1 | 4 | 7 | 1 | 2 |
| TX | 571 | 2 | 3 | 0 | 0 | 0 | 0 | 1 | 2 | 3 | 5 | 2 | 2 |
| TX | 574 | 2 | 3 | 0 | 0 | 1 | 1 | 0 | 3 | 3 | 7 | 1 | 2 |
| TX | 578 | 2 | 3 | 0 | 0 | 1 | 1 | 1 | 2 | 4 | 6 | 1 | 1 |
| TX | 584 | 2 | 3 | 0 | 0 | 1 | 1 | 1 | 2 | 4 | 6 | 1 | 1 |
| TX | 588 | 2 | 3 | 0 | 0 | 0 | 0 | 1 | 2 | 3 | 5 | 1 | 1 |
| TX | 591 | 2 | 3 | 0 | 0 | 1 | 1 | 0 | 1 | 3 | 5 | 1 | 1 |
| TX | 592 | 2 | 3 | 0 | 0 | 1 | 1 | 1 | 2 | 4 | 6 | 1 | 1 |
