# Supplementary material for: Sheep breed-specific response to environment challenge against Haemonchus contortus and effect on immuno-hematological parameters
Source: Vet Res Commun. 2026 Jun 6;50(5):372. doi: 10.1007/s11259-026-11304-2 (PMC13242422; doi:10.1007/s11259-026-11304-2)

Supplementary information 3. Relative quantification of gene expression in the fundic abomasum of White Dorper (DO), Santa Inês (SI) and Texel (TX) lambs naturally infected with *H. contortus*. The p-values located in the up and right side of figures were obtained by Tukey test. The ”t” letter in the y-axis title indicated transformed data.


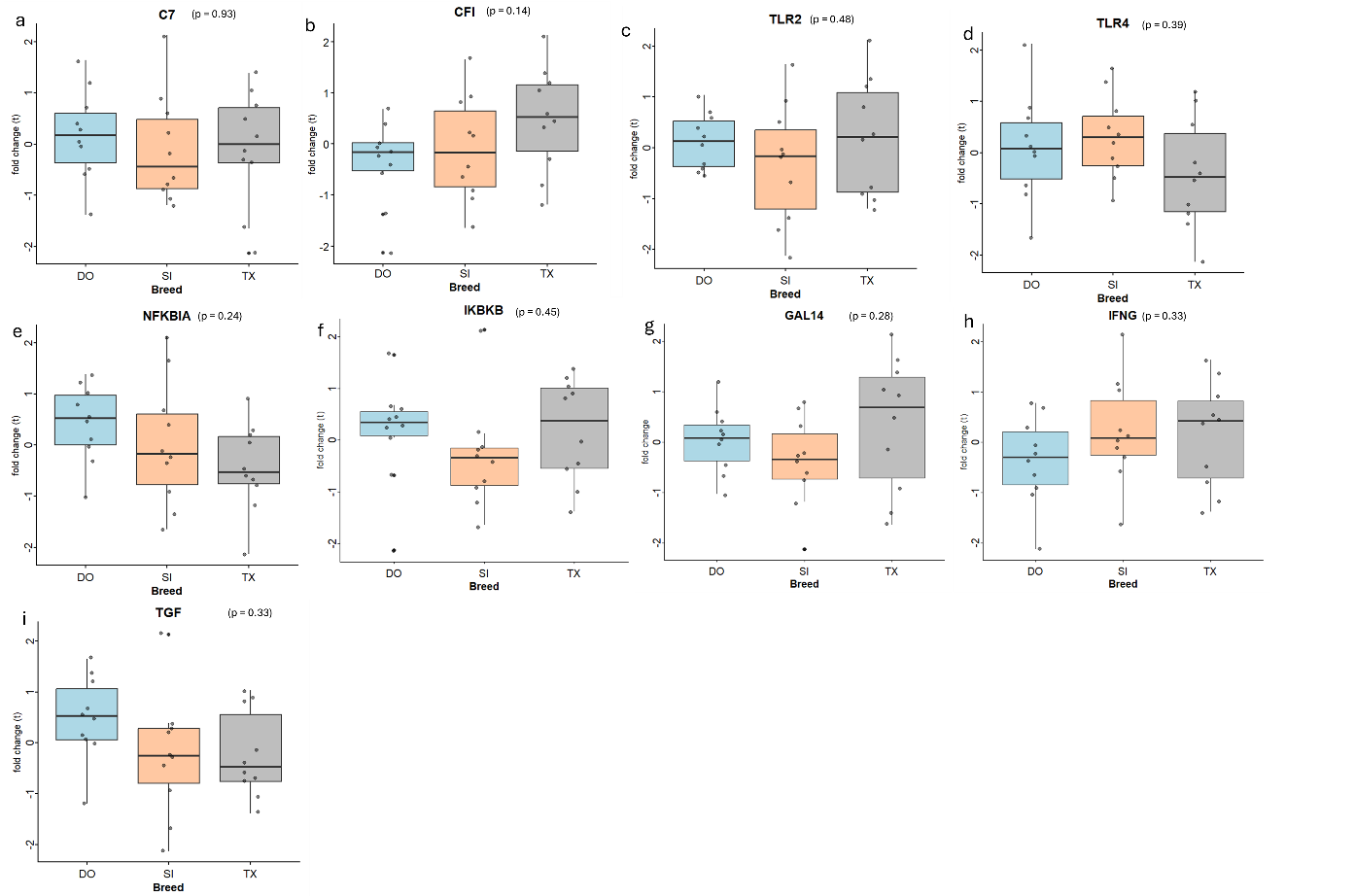

Supplement: Supplementary file 4 — Supplementary Material 4 [file 11259_2026_11304_MOESM4_ESM.docx]
